# 

**Published:** 1887-11

**Authors:** 


### TABLE OF CONTENTS.

ORIGINAL AND SELECTED ARTICLES.

Man as an Object of Zoology, by S. W. Stiles, M. D., Georgia .......... 441
A Case of Douloureux of Twelve Years' Standing Treated by Large Doses of the Salicylates, with Marked Success, by Francis X. Dercum, M. D .......... 443
Rectal Surgery Made Easy .......... 446
Report and Remarks on a Series of Two Hundred Cataract Operations, by C. A. Bucklin, A.M., M.D., New York .......... 450
A Clinical Study of Purulent Ophthalmia, by John T. Oldham, M. D., Louisville, Ky .......... 453
The Revolution in the After Treatment of Cataract Operations, by Julian J. Chisholm, M.D., Baltimore, M. D .......... 454
A Remedy for Lumbago .......... 456

ABSTRACTS AND GLEANINGS.

Oil of Erigiron .......... 457
Diagnosis and Treatment of Typhoid Fever; Red Wine in "Bed-Wetting." .......... 458
Uses of Menthol .......... 459
Report of the English Commission on Pasteur's Hydrophobia Inoculation .......... 460
Chloroform and Ether .......... 461
Physiological Action of Cocaine; Creosote in Phthisis .......... 462
Tartar Emetic in Labor; The Treatment of Dysentery; Dental Anæsthesia .......... 463
Salicylate of Sodium; The Value of Lantanin in Malaria; Extraction of a Splinter of Iron from the Vitreous Humor by Means of an Electro-Magnet .......... 464
"The Treatment of Ulcus Cruris by a New and Successful Method of Dr. Unna;" Antifebrin .......... 465
Marriage by Syphilitics—When Safe .......... 466
Stenocarpine the New Substitute for Cocaine .......... 467
A New Era in Cataract Extraction; A Test for Arsenic in Wall-Paper .......... 468
Method of Inducing Respiration; Whitlow; Antipyretics .......... 469
Neutral Hydrochloride of Quinine; Bromide of Potassium in Ptyalism of Pregnancy; Antipyrin in Bilious Headache; Treatment of Acute Miliary Tuberculosis by Iodide of Sodium .......... 470

SCIENTIFIC ITEMS.

Theine; Electrified Ice Cream; Something New About Bees .......... 471

Interesting Cure of Insanity; The Skeleton in the Cupboard .......... 472

PRACTICAL NOTES AND FORMULÆ.

Soothing Mixture for Consumptives; Barium Chloride in Vascular Diseases; Pruritus of the Female Genitals; Treatment of Hepatic Conjestion .......... 473
Syrup Trifolium Comp. in Syphilis; An Enema for Convulsions of Children; Treatment of Flatulent Dyspepsia; For Mammary Inflammation .......... 494

EDITORIALS AND MISCELLANEOUS.

Death of Dr. G. W. Owen; Editorial Brevities 475
Southern Medical College Opening Exercises. 476
Ninth International Medical Congress .......... 477
Special Notes .......... 483

